# Early Senescence and Leukocyte Telomere Shortening in SCHIZOPHRENIA: A Role for Cytomegalovirus Infection?

**DOI:** 10.3390/brainsci8100188

**Published:** 2018-10-18

**Authors:** Corona Solana, Diana Pereira, Raquel Tarazona

**Affiliations:** 1 Centro Hospitalar Psiquiatrico de Lisboa, 1700-063 Lisboa, Portugal; dmr_pereira@hotmail.com; 2 Immunology Unit, University of Extremadura, 10003 Caceres, Spain

**Keywords:** schizophrenia, leukocyte telomere length, cytomegalovirus, inflammation, early senescence

## Abstract

Schizophrenia is a severe, chronic mental disorder characterized by delusions and hallucinations. Several evidences support the link of schizophrenia with accelerated telomeres shortening and accelerated aging. Thus, schizophrenia patients show higher mortality compared to age-matched healthy donors. The etiology of schizophrenia is multifactorial, involving genetic and environmental factors. Telomere erosion has been shown to be accelerated by different factors including environmental factors such as cigarette smoking and chronic alcohol consumption or by psychosocial stress such as childhood maltreatment. In humans, telomere studies have mainly relied on measurements of leukocyte telomere length and it is generally accepted that individuals with short leukocyte telomere length are considered biologically older than those with longer ones. A dysregulation of both innate and adaptive immune systems has been described in schizophrenia patients and other mental diseases supporting the contribution of the immune system to disease symptoms. Thus, it has been suggested that abnormal immune activation with high pro-inflammatory cytokine production in response to still undefined environmental agents such as herpesviruses infections can be involved in the pathogenesis and pathophysiology of schizophrenia. It has been proposed that chronic inflammation and oxidative stress are involved in the course of schizophrenia illness, early onset of cardiovascular disease, accelerated aging, and premature mortality in schizophrenia. Prenatal or neonatal exposures to neurotropic pathogens such as Cytomegalovirus or Toxoplasma gondii have been proposed as environmental risk factors for schizophrenia in individuals with a risk genetic background. Thus, pro-inflammatory cytokines and microglia activation, together with genetic vulnerability, are considered etiological factors for schizophrenia, and support that inflammation status is involved in the course of illness in schizophrenia.

## 1. Introduction

Schizophrenia is a severe, chronic mental disorder, with a heterogeneous genetic and neurobiological background, characterized by delusions, hallucinations and thought disorder, that causes an important impairment in social and vocational functioning. Current treatment consists largely in the use of antipsychotic drugs combined with psychological and social support, but no cure is currently available, mostly due to the poor understanding of the disease pathogenesis [1,2,3,4]. The etiology of schizophrenia is multifactorial, involving genetic and environmental factors [5].

It has been suggested that several genes can be associated with schizophrenia [6,7,8], including those associated with synaptic function and neurobehavioral phenotypes [9]. Accumulating data using new genomic approaches in combination with extensive neuroimaging collections of brain phenotypes is contributing to the definition of new putative schizophrenia risk genes [10,11] and support the interest of genetically-based patient stratification in patient subgroups sharing deficits in particular biological pathways [12] or the use of common genetic variants to predict endophenotypes such as spatial visualization [13].

In relation with the environmental factors, epidemiological and serological studies in the last decade have highlighted the significance of chronic infections by several pathogens, including viruses (Cytomegalovirus, CMV, and other herpesviruses) and parasites, such as Toxoplasma gondii (T. gondii), in the pathogenesis and clinical evolution of schizophrenia [14,15].

Schizophrenia patients show higher mortality rates than those who died without schizophrenia [16,17,18,19,20]. In a recent meta-analysis, it has been estimated that schizophrenia is associated with 13–15 years of potential life lost and with a life expectancy of 60 years for men and 68 years for women [21]. It has been recently suggested that schizophrenia might be considered as a syndrome of “accelerated aging” [22] or as a “segmental progeroid syndrome” [23].

In this review, we present and discuss recent evidences supporting the role of infection by CMV and other pathogens and inflammation in telomere shortening and accelerated senescence in schizophrenia patients.

## 2. CMV Is a Major Driver of Immunosenescence

Several biological parameters have been defined as hallmarks of ageing [24]. These hallmarks include the age-associated deterioration of the immune response, also termed immunosenescence, and the existence of age-associated low-grade inflammation usually known as inflamm-ageing [25]. CMV infection is associated with immunosenescence and the accumulation of highly differentiated T cell clones [26,27] characterized by the production of pro-inflammatory cytokine [28,29,30,31,32,33]. One of the major hallmarks of immunosenescence is leukocyte telomere shortening [34].

Telomeres are DNA and protein structures present at the ends of chromosomes to prevent the loss of coding genetic material during cell replication. Telomeres shorten with each cell division and shortened telomeres induce a DNA damage response leading to a growth arrest and, eventually, triggering replicative senescence [35,36,37,38]. Telomerase, which it is normally expressed in pluripotent and adult stem cell, compensates for telomere attrition by the addition of TTAGGG repeats onto chromosome ends [39,40,41,42]. Telomerase-deficient mice show premature aging phenotypes, providing the first evidences that telomere attrition is a determinant of longevity and is at the origin of different age-related pathologies [43,44,45].

Telomere attrition occurs in normal tissues at an estimated rate of 50 to 150 base pairs per cell division, resulting in a gradual decrease in median telomere lengths with increasing age [46]. Circumstances associated with replicative stresses enhance telomere shortening secondary to the increased mitotic activity. Thus, chronic infection by different pathogens, in particular chronic viruses, accelerate leukocyte telomere length (LTL) attrition [47,48]. In addition, the production of reactive oxygen species associated with chronic inflammation, that can also be produced in response to chronic infection, several drugs and toxic products, or radiation exposure, also cause DNA damage and telomere loss [49]. Finally, inheritable gene mutations affecting the telomerase or the telomere protein complex, resulting in decreased telomere lengths (short telomere syndromes, STSs) [50,51]. These syndromes are characterized by severe alterations in hematopoiesis (bone marrow failure) and in organs with high cell turnover, such as skin (dyskeratosis), gastrointestinal tract (esophageal stenosis, enterocolitis, celiac-like enteropathy) and lungs (idiopathic pulmonary fibrosis) [50,51].

### 2.1. CMV and Leukocyte Telomere Shortening in Healthy Ageing

In humans, telomere studies have mainly relied on measurements of LTL, which reflects telomere length (TL) in other somatic cells. LTL displays high variation across individuals, a phenomenon already observed in newborns and that has been related to different factors such as sex, ethnicity, or parental age at conception (reviewed in [52]). LTL is considered a biomarker of human ageing, and it is generally accepted that individuals with short LTL are biologically older than those with longer ones. However, recent evidences suggest that short telomeres increase the risk of age-related diseases and play a role in the development of these diseases. Although the causes of age-associated frailty are multiple, telomere shortening, immunosenescence and inflamm-ageing have been implicated in its pathophysiology.

Peripheral blood T lymphocytes that have undergone a process of replicative senescence (immunosenescence) are characterized by loss of proliferation, telomere shortening, and by the decreased expression of CD28 and CD27 co-stimulatory molecules and the expression of CD57 [34,53,54,55,56]. These hallmarks of immunosenescence increase with healthy ageing and also in clinical situations associated with chronic activation of the immune response and an accumulation of CD28−CD27−CD57+ T cells occurs during normal aging and chronic-antigen stimulation [25,57,58,59]. Senescence and telomere shortening is observed in particular in CMV infection, characterized by accumulation of cells with a more differentiated state and shorter telomeres than in other virus infections, such as human immunodeficiency virus (HIV), Epstein-Barr virus (EBV), and hepatitis C virus (HCV) [47,48], indicating that CMV is a major driver of immunosenescence.

LTL correlates with lymphocyte telomere length and with the amount of highly differentiated T cells. In addition, telomere shortening is more rapid in CMV-seropositive individuals, indicating that CMV infection induces a strong decrease in T cell telomere length [60]. CMV seropositivity and CMV IgG antibodies correlate not only with LTL but also with telomerase activity [61] The telomere lengths in total and CMV-specific T cells are shorter in old compared to young individuals, although the accumulated end-stage cells are multifunctional T cells and do not have the shortest telomeres [31]. CMV seropositivity and increased total pathogen burden level are significantly associated with shorter telomere length among females [62]. Infection by CMV and other herpes viruses (herpes simplex virus type 1 (HSV1), and human herpesvirus 6) are associated with greater LTL attrition, and higher IgG anti-CMV were also associated with shorter LTL after 3-year follow-up [63]. In a recent longitudinal analysis of healthy individuals (age range 21 to 88 years), with an average follow-up of 13 years (7–19 years) follow-up, age-related telomere attrition, elevated pro-inflammatory cytokines and anti-CMV IgG levels were observed, although no significant correlations among these inflamm-ageing and immunosenescence related parameters were observed, indicating the complexity of the immune aging processes [64].

### 2.2. CMV Seropositivity and Inflammation

Since the original demonstration that CMV seropositivity was associated with phenotypic and functional alterations of T-cell immunity similar to those found in ageing [27], and that CMV infection induces a strong decrease in T cell telomere length [60], cumulative evidences have extensively documented that CMV infection is characterized by the expansion of CD28/CD27 negative or CD57+ senescent T cells, with short telomeres and high production of pro-inflammatory cytokines [25,26,34,54,60,65,66,67,68,69,70,71,72]. The analysis of CMV in frailty has shown that the frequency of CMV reactivation is associated with aging and ongoing frailty [73], while CMV-seropositivity is not associated with pre-frailty in very old subjects [74].

The possible relationship between CMV infection with psychological stress has been recently highlighted in a recent study showing that early-life adverse and stressful events, such as parental loss or low socioeconomic status, increase the risk of CMV infection. The authors also propose that CMV-driven immunosenescence and inflammation underlie the association between early life adverse and the long-term health consequences such as cardiovascular disease and type 2 diabetes observed in these individuals [75].

## 3. Telomere Length in Age-Associated Diseases

LTL shortening has been proposed to be a primary molecular cause of ageing in healthy individuals [24,42,76,77,78]. Recent evidences provide direct evidences supporting a direct role for inflammation in telomere shortening [64,79]; indirect evidences have clearly shown a strong association between these two parameters in healthy ageing and in different clinical circumstances, including mental diseases (for review see [49]). Telomere erosion has been shown to be accelerated not only by chronic viral infections (see Section 2.1), but also by different factors, including some medical illnesses [50,80,81,82,83], and other environmental factors associated with oxidative stress and chronic inflammation, such as cigarette smoking [84] and chronic alcohol consumption [85] or by psychosocial stress such as childhood maltreatment [86].

In addition, different research groups have reported that many detrimental traits and potentially harmful environmental factors are associated with short telomeres in humans. Accordingly, comparatively short LTL is considered a marker not only of ageing but also of poor health, regardless of the person’s age [87,88,89,90], which predicts all-cause mortality [91]. A significant LTL shortening has also been found across psychiatric disorders [92,93,94].

LTL is a predictor of coronary heart disease events in middle-aged, high-risk men, and reduced LTL is associated with all-cause mortality in patients with stable cardiovascular disease [95,96,97,98,99]. Furthermore, acute myocardial infarction and reperfusion accelerate immunosenescence in cytomegalovirus-seropositive patients [100]. LTL attrition also associates with obesity [101,102] and with the presence and number of diabetic complications in type 2 diabetes patients [103,104,105,106,107,108,109]. It has been recently shown that reducing obesity may reduce the risk of diabetes complications associated with shorter LTL, at least in some Chinese population [110].

Previous studies have shown that many psychiatric illnesses are associated with shortened telomeres [93,94], including major depression [111], chronic mood and anxiety disorders [112] or schizophrenia [113]. In major depressive disorder, LTL is proportional to lifetime exposure to the disease, suggesting that LTL attrition is not related to the origin of this disease but on the contrary it is associated with oxidative stress and inflammation [114], and with accelerated ageing [114,115,116,117]. Further studies are needed to clarify the possible role of TL shortening in the clinical outcome of major depression. The observation that TL shortening is associated with pharmacological treatment [118] and the finding that short LTL is also an index of poor response to selective serotonin reuptake inhibitor (SSRI) treatment [119] might reflect the severity of depression in group of patients with shorter telomeres. Metabolic stress, pro-inflammatory cytokines and metabolic alterations, and lifestyle factors, e.g., cigarette smoking, are also important mediators of the association between depressive and anxiety disorders and LTL [120].

Thus, the traditional view of LTL as a passive biomarker of human ageing is under revision as new evidences suggest that the interplay between evolutionary forces and TL might result in distinct health outcomes. While individuals with short telomeres might have a higher risk of diseases related to restricted cell proliferation and tissue degeneration, including cardiovascular, metabolic or mental diseases, those with long telomeres might have an increased risk of diseases related to increased proliferative growth, including cancer [52].

## 4. Infection by CMV, T. Gondii and other Pathogens in Schizophrenia

**Figure 1:**
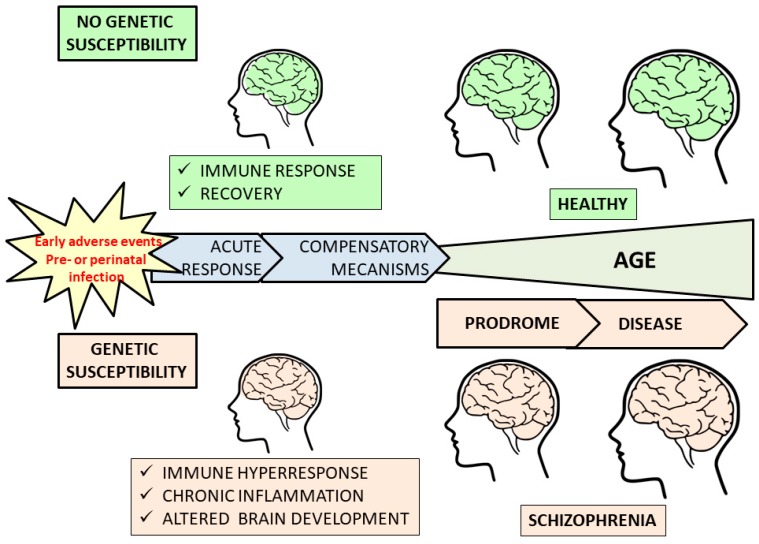
**Schematic representation of the possible role of prenatal infections in schizophrenia pathophysiology.** Exposure to neurotropic pathogens such as cytomegalovirus (CMV), other herpes viruses or T. gondii, or early adverse or stressful events has been proposed as environmental risk factors for schizophrenia in individuals with genetic susceptibility. Thus, maternal immune response to infection during pregnancy induce the production of pro-inflammatory cytokines, such as interleukin (IL)-6, IL-1, tumor necrosis factor (TNF)-α and interferon (IFN)-γ, which activate placenta, fetal blood vessel and meninges. Inflammation may transiently alter brain development that, in the absence of genetic susceptibility, recovers in a short period of time. However, in the presence of genetic susceptibility to schizophrenia, the fetal meninges and blood vessels are hyper-responsive to these cytokines by secreting more cytokines and chemokines, the compensatory mechanisms (e.g., gene expression changes induced by IFN-γ) are exhausted and finally results in chronic inflammatory responses interfering with brain development. The inflammation status also influences postnatal brain development and the course of illness that after a prodrome phase finally results in schizophrenia. (Brain icon was obtained from: https://www.wpclipart.com/medical/anatomy/brain/brain_icon.png.html).

Maternal or neonatal exposure to neurotropic pathogens, such as herpes viruses and T. gondii, has been proposed as an environmental risk factor for schizophrenia in individuals with a risk genetic background (Figure 1). The measurement of pathogen-specific immunoglobulin is a marker of previous contact with each pathogen (IgM for first and IgG for longer term contact), whereas IgG levels are associated with repeated reactivation of the pathogens. Serostatus and titer of IgG against several herpes viruses and T. gondii have been studied in schizophrenia patients, but the results are still inconclusive.

It has been shown that chronic maternal infection with T. gondii or CMV can affect neonatal innate immunity and it has been proposed that chronic infections by these pathogens contribute to increased risk for psychosis [121]. Results from other groups also support that the increase in antibody levels to several viruses is predictive of an 18–34% increase in the risk of developing schizophrenia [122] or that anti-CMV and anti-HSV1 antibody levels are significantly increased in schizophrenia patients [123]. However, others have not found significant differences in antibody titers against the pathogens studied, and therefore do not support the hypothesis that increased exposure to neurotropic pathogens after birth is associated with schizophrenia [124,125]. Other evidences demonstrate that CMV infection is associated to poorer markers in the evolution of schizophrenia. Thus, CMV-seropositive schizophrenia patients show higher negative symptoms scores [125], low California Verbal Learning Test (CVLT) scores, decreased hippocampal volume, and poorer episodic verbal memory [126] than those CMV-seronegative patients. In addition, the risk for suicide in persons with serious mental illness, including schizophrenia, bipolar disorder, or major depression, has been shown to be associated with elevated levels of IgM antibodies to both T. gondii and CMV [127] or with elevated levels of antibodies to CMV [128] indicating that, even if these pathogens are not directly the cause of schizophrenia, they are involved in the pathogenesis, evolution, and complications of the disease.

## 5. Schizophrenia, Inflammation, and Early Senescence

**Figure 2:**
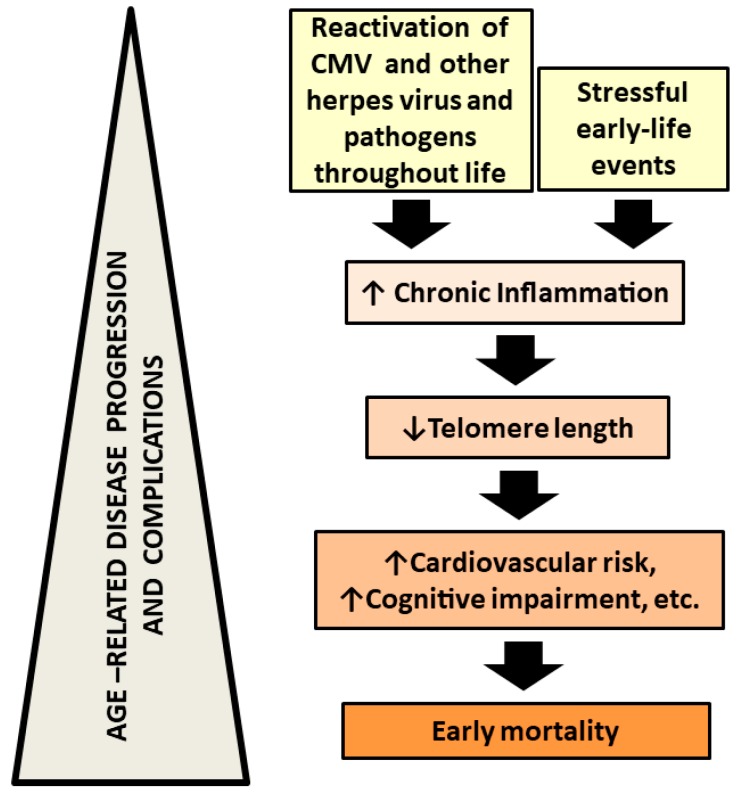
**Possible mechanisms involved in schizophrenia associated early ageing.** Reactivation of pathogens, such as Cytomegalovirus (CMV), involved in chronic inflammation together with other stressful events and genetic vulnerability can be considered etiological factors for schizophrenia and other mental diseases. Chronic inflammation induces telomere shortening that is related to increased cardiovascular risk and cognitive impairment altogether involved in early mortality in schizophrenia patients.

Schizophrenia and other mental disorders are associated with persistently high rates of morbidity and mortality, despite the widespread use of treatments. Although the causal mechanisms of the early mortality in schizophrenic patients are still poorly understood and require further studies, schizophrenia has been associated with oxidative stress and chronic inflammation, which may be associated with the early onset of inflammatory age-associated syndromes such as cardiovascular disease or type 2 diabetes, accelerated ageing, and premature mortality in schizophrenia [129] (Figure 2).

### 5.1. Schizophrenia and Inflammation

Cumulative evidences from epidemiological, genetic and peripheral biomarkers in schizophrenia and mood disorder patients point to a dysregulation of innate and adaptive immune systems in the disease, and support that these immune abnormalities contribute to disease symptoms, at least in a subpopulation of patients [130]. It has been suggested that immune activation with a shift to a pro-inflammatory state of the cytokine network in response to still undefined environmental agents, herpesviruses infections, or microbiome, can be involved in the pathogenesis and pathophysiology of this mental illness. An increased level of pro-inflammatory markers in both peripheral and cerebral systems has been consistently documented in schizophrenia, supporting that a concomitant process of inflammatory activity is involved in the progression of schizophrenia [131,132]. The analysis of oxidative and inflammatory markers in psychosis shows that people with schizophrenia have increased pro-inflammatory and pro-oxidative status and the results suggest that greater inflammation and oxidative stress might lead to poorer outcomes in patients with first episodes of early onset psychosis [133,134].

### 5.2. Leukocyte Telomere Shortening in Schizophrenia

The exact causes and mechanisms involved in early cellular senescence in schizophrenia and its possible significance in the pathogenesis and clinical evolution of the disease are still poorly understood. It has been suggested that LTL shortening is involved in this process. Nevertheless, there are discrepancies among the results of different groups, with studies reporting that LTL in schizophrenia can be shorter [135,136,137,138], similar [139,140], or even longer [141,142] than in controls. Furthermore, a recent study has also reported short telomeres in patients at ultra-high risk for psychosis [143]. There are different reasons that can help to explain these discrepancies, going from the different techniques used to study LTL, to epidemiological parameters (such as age, gender, ethnicity, or style of life) or clinical aspects (such as disease progression, treatment response, or the presence of comorbidities) that affect LTL [94,113,144,145] and could be acting as confounding factors in the different studies. Of particular interest is the finding that telomere length correlates with positive scores using the Positive and Negative Syndrome Scale (PANSS) [142]. However, despite these limitations, most recent studies and meta-analysis support the presence of telomere shortening in schizophrenia patients.

Thus, decreased LTL and increased levels of C-C Motif Chemokine Ligand 11 (CCL11), which crosses the blood-brain barrier and is involved in neuroinflammation, are related with reduced grey matter volume and a longer duration of illness, consistent with the hypothesis that schizophrenia is associated with a pathological accelerated ageing, leading to impaired outcomes in these patients [146]. Telomere shortening also has a detrimental effect on brain size in healthy individuals, but the association between these parameters is significant only for those older than 50 years [147], while in schizophrenia patients this relationship was observed at 36 years [146]. In agreement with these results, another recent study has shown that the diagnosis of schizophrenia, more than other possible confounding parameters such as gender, age, cigarette smoking or alcohol drinking, is the most important condition responsible for LTL shortening in these patients, and that major differences in LTL shortening were mainly observed in patients younger than 50 years, while no significant differences were observed in the group of older subjects [148].

The reasons underlying the phenomenon of telomere shortening in schizophrenia are poorly understood and there is likely a combination of several genetic, environmental, and psychosocial parameters in this process. Although schizophrenia is associated with different genetic alterations, there are no evidences suggesting that they are directly involved in telomere shortening. A significant decrease in telomerase activity among individuals with schizophrenia compared to unaffected individuals has been defined in schizophrenia patients [149]. However, no reports on telomerase alterations, which have been defined in several genetic diseases with disease phenotypes that overlap those normally acquired with ageing [150,151], or in DNA telomere point mutations that accelerate shortening leading to premature ageing [152], have been defined.

On the contrary, infection by CMV, a well-known driver of immunosenescence, (see Section 2), which includes leukocyte telomere shortening and the expansion of T and Natural Killer (NK) cell subsets characterized by the production of pro-inflammatory cytokine, has been postulated to be involved in the pathophysiology and clinical evolution of schizophrenia in genetically susceptible individuals. In a similar way, a pro-inflammatory state has been associated with telomere shortening in different age-associated clinical syndromes such as cardiovascular disease, type 2 diabetes, or neurodegenerative conditions (reviewed in [89]), and also in schizophrenia [146]. The relationship between CMV infection, pro-inflammatory state and telomere shortening in age-associated diseases, that is also observed in schizophrenia, can also be directly or indirectly responsible for the high incidence of comorbidities and premature mortality in schizophrenia patients [22,94].

It has also been suggested that psychological stress plays a role in LTL shortening in schizophrenia. In studies with a small number of patients and controls, significant telomere shortening is observed only in female patients with perceived childhood trauma [153] or in situations of not perceived stress such as in cases of social isolation and loneliness [154], have been postulated to be involved in telomere shortening and accelerated ageing in specific groups of schizophrenia patients. These hypotheses are mutually exclusive. Thus, it has been shown that early-life adverse and stressful events increase the risk of CMV infection [75]. Therefore, the linkage between psychological stress and CMV infection, and the subsequent immunosenescence and inflammation underlie the association between early life adverse and long-term health consequences such as cardiovascular disease and type 2 diabetes observed in these individuals.

Telomere length has been studied in selected brain areas in schizophrenia. This disease is characterized by an initial, rapid rate of grey matter loss that slows in middle life, followed by an appearance of a progressive white matter deterioration [155]. The results show a significant reduction of telomere length in superior temporal gyrus white matter of patients with schizophrenia as compared to controls, with no alterations in telomere length in medial frontal gyrus grey and white matter and in superior temporal gyrus grey matter, supporting cell senescence in white matter temporal brain tissue in these patients [156]. Furthermore, schizophrenia patients show accelerated brain ageing (based on the analysis of grey matter density maps), which occurs mainly during the first year after disease onset [157].

Based on the above observations, it can be suggested that schizophrenia is linked with accelerated cellular ageing [94,157], possibly involving telomere shortening [135,136,137,138,146,148,156].

## 6. Concluding Remarks

The observation that schizophrenia patients show higher mortality rates than people without schizophrenia has evidenced that one of the major complications of this mental illness is premature senescence. The etiology of schizophrenia is multifactorial and involves genetic and environmental factors. Pre- or perinatal infection by pathogens such as CMV and other herpesviruses and T. gondii, and a dysregulation of both innate and adaptive immune systems resulting in immunosenescence and inflammation, have been postulated as environmental factors involved in the pathogenesis of this disease, with a specific effect promoting short LTL shortening and accelerated cellular aging. These factors are also associated with the onset of age-associated diseases such as cardiovascular disease and can be responsible, at least in part, for accelerated ageing and premature mortality in schizophrenia [129]. Although the effect of telomere shortening on the pathogenesis and co-morbidities of human mental disorders should be regarded with caution, further studies to better understand the environmental factors contributing to telomere length attrition will contribute to better understand the pathophysiology of schizophrenia and other mental disorders and to design novel therapy strategies targeting pathogen infection and dysregulated immunological and inflammatory responses in these patients [158].
